# Assessing cerebral arterial pulse wave velocity using 4D flow MRI

**DOI:** 10.1177/0271678X211008744

**Published:** 2021-04-14

**Authors:** Cecilia Björnfot, Anders Garpebring, Sara Qvarlander, Jan Malm, Anders Eklund, Anders Wåhlin

**Affiliations:** 1 Department of Radiation Sciences, Umeå University, Umeå, Sweden; 2 Department of Clinical Science, Neurosciences, Umeå University, Umeå, Sweden; 3 Umeå Center for Functional Brain Imaging (UFBI), Umeå University, Umeå, Sweden

**Keywords:** Atherosclerosis, arterial stiffness, arteriosclerosis, magnetic resonance imaging, neurovascular dysfunction

## Abstract

Intracranial arterial stiffening is a potential early marker of emerging cerebrovascular dysfunction and could be mechanistically involved in disease processes detrimental to brain function via several pathways. A prominent consequence of arterial wall stiffening is the increased velocity at which the systolic pressure pulse wave propagates through the vasculature. Previous non-invasive measurements of the pulse wave propagation have been performed on the aorta or extracranial arteries with results linking increased pulse wave velocity to brain pathology. However, there is a lack of intracranial “target-organ” measurements. Here we present a 4D flow MRI method to estimate pulse wave velocity in the intracranial vascular tree. The method utilizes the full detectable branching structure of the cerebral vascular tree in an optimization framework that exploits small temporal shifts that exists between waveforms sampled at varying depths in the vasculature. The method is shown to be stable in an internal consistency test, and of sufficient sensitivity to robustly detect age-related increases in intracranial pulse wave velocity.

## Introduction

Cerebral arterial stiffness is implicated in a range of neurological diseases among the elderly, including cerebral small vessel disease,^1^ mild cognitive impairment and Alzheimer’s disease.^2,3^ Arterial stiffening occurs with biological aging that causes structural changes such as breakdown of elastin, increases in collagen content, as well as thickening of the arterial wall.^4^ Such structural remodeling is aggravated by hypertension^5^ and linked to the development of atherosclerosis.^6^

The ways through which alterations to the structure of cerebral blood vessels could lead to brain damage are not completely known but could involve neurovascular dysfunction and blood-brain barrier disruption,^7^ potentially as a consequence of excessive downstream exposure of cardiac-related pulsatility.^8^ Another potential mechanism is a stiffness-induced dysfunction of vascular pulsations driving glymphatic flow.^9,10^ Importantly, different vascular segments may be differentially expressing pathological changes,^11,12^ causing uncertainty regarding how closely extracranial measures reflect intracranial pathology, stressing the need for “target-organ” measurements.

From a fluid mechanics perspective, the alterations to the vessel wall influence the transmission of blood flow and pressure pulsatility. Notably, arterial wall stiffening increases the pulse wave velocity (PWV), the rate at which the pressure pulse propagates through the vasculature.^13^ The current gold standard, carotid-femoral PWV, is sensitive to the velocity of the pressure pulse in the aorta and has been used to demonstrate links between vascular stiffness and microvascular brain damage,^14^ beta-amyloid deposition^15^ and cognitive decline.^16^

Cardiac-gated 4D flow MRI offers time resolved velocities in all three spatial directions and is increasingly used to assess blood flow in the brain.^17^ 4D flow MRI with a reconstructed temporal resolution of ∼10 ms has been used to demonstrate internal carotid stiffness increase in AD.^18^ 4D flow MRI potentially also allows estimating PWV in the distal intracranial vasculature. However, velocity waveforms from single cross-sections in small arteries are typically noisy, illustrated by the need for averaging of multiple cross-sections when measuring flow velocity in distal cerebral branches.^19,20^ Inspired by such multi-cross-section averaging, we hypothesized that minute temporal shifts between a large amount of intracranial arterial cross-sections would allow estimating a PWV if the path distance from each cross-section to the root of the arterial tree is known. This bears similarity with a concept developed for multi-cross-section analyses for aortic PWV,^21^ with the additional complexity of considering the rapidly branching structure of the intracranial vascular tree.

In this study, we investigate this concept and propose a 4D flow MRI method that simultaneously considers time-resolved velocity waveforms from all detected vessel cross-sections of the cerebrovascular tree to estimate a global intracranial PWV. We deploy the measurements in healthy young and elderly individuals to describe the effect of age on our derived PWV measure.

## Materials and methods

### Participants

The present study combines an elderly^19^ and a young^22^ cohort scanned using the same scanner and sequence. The young cohort consisted of 10 healthy volunteers (mean age 37 years, range 29–53 years, three women), and the elderly cohort consisted of 35 participants (mean age 79 years; range 70–91 years; 20 women). The elderly cohort was originally recruited in 2007 and then consisted of 50 subjects aged 60–82 years without psychiatric or neurological disorders and without signs of advanced atherosclerotic disease.^19^ A 4D flow MRI was added to their 10-year follow-up scan and 35 individuals successfully completed the follow-up examinations. The elderly participants were examined by a neurologist and the following diseases were now observed among the participants: cerebrovascular disease, *N* = 5; Alzheimer’s disease, *N* = 2; Lewy body dementia, *N* = 1; ischemic heart disease, *N* = 2; diabetes mellitus, *N* = 2; and atrial fibrillation, *N* = 3. The number of cases for any particular disease was considered too small to examine for potential differences in PWV. Instead, in a post-hoc analysis we focused on the relationship between PWV and age, whereby elderly cases with any of the aforementioned diseases were excluded, leaving a healthy elderly sample of *N* = 21. In addition to the recorded diseases, the following vascular risk factors were noted among the elderly: hypertension, *N* = 23; hyperlipidemia, *N* = 14; and ever smoker *N* = 7.

The study was conducted according to the Helsinki ethical principles with obtained approval from the ethical review board of Umeå University (211/440-31M; 2017/253-31). Written and oral informed consent was obtained from all participants.

### MRI protocols

All participants underwent 4D flow MRI acquisitions on a 3T scanner (Discovery MR 750; GE Healthcare, Milwaukee, Wisconsin) with a 32-channel head coil. No contrast injection was used. Acquisitions with full-brain coverage were performed utilizing PC-VIPR,^23^ a highly undersampled radial projection sequence able to acquire time-resolved flow rates in all three spatial directions with a scan duration of approximately 9 min. Data collection was performed with the following scan parameters; five-point velocity encoding, velocity encoding sensitivity ( $v_{\mathrm{enc}}$ ): 110 cm/s; repetition time/echo time (TR/TE): 6.5/2.7 ms; flip angle: 8°; radial projections: 16,000; temporal resolution: 20 frames per cardiac cycle; acquisition matrix: 300 × 300 × 300 voxels; imaging volume: 22 × 22 × 22 cm; reconstruction matrix: 320 × 320 × 320 voxels with a 0.69 mm isotropic voxel size.

### 4D flow MRI processing

From the collected 4D flow MRI data a magnitude volume and velocity volumes in all three directions were reconstructed. To highlight vascular structures containing flow, the magnitude and velocity images (temporal averages) were combined to form a complex difference (CD) angiogram, here using the relationship: $\;CD=m\;*\;\sin\left(\theta \right)$ , where m is the magnitude and $\theta =\;\pi \sqrt{v_{x}^{2}+v_{y}^{2}+v_{z}^{2}}/v_{\mathrm{enc}}$ . The value of θ was truncated to be in the range $\theta \in \left[0\;\pi /2\right]$ . The CD volume was further processed with a vessel enhancing filter^24^ (*τ* = 1) to improve visibility of the small arteries prior to undergoing a binarization based on a global intensity threshold (at 2.5% of the global maximum). Skeletonization and centerline processing was performed to obtain a branch representation of the vasculature.^25^

Velocity data was obtained by a previously developed algorithm, specifically devised for intracranial 4D flow MRI data.^26^ Cross-sections perpendicular to the vessel direction were automatically positioned along all voxels in the centerline representation. The direction was estimated from three consecutive centerline voxels centered around the analyzed centerline position. Prior to vessel segmentation, each two-dimensional cross-section was linearly interpolated with a factor of two to increase resolution. A region of interest (ROI) was then obtained by applying a local intensity threshold in the CD cross-section image (here set at 50% of the peak value within the vessel). The obtained ROI was then used to calculate average *x*-, *y*-, and *z*-velocities used as a velocity vector for each time frame in the cardiac cycle. A dot product between the velocity vector and the direction of the vessel given by the centerline provided the final velocity estimate. This resulted in time-resolved velocities for all captured cross-sections in the cerebral vasculature. As a preprocessing step, the temporal mean velocity was removed from each waveform and the resulting curve was scaled by the reciprocal of the standard deviation ( $\mathrm{scaling}={SD}^{-1})$ . Flow velocity curves were not calculated for junction points.

In this study, the desired vascular tree consisted of the arteries in the anterior cerebral circulation. To remove vessels of no interest, branch identities representing bridging veins and venous sinuses, as well as the posterior cerebral circulation arteries were manually removed by visual inspection in maximum intensity projections of labeled centerline branches. This entailed cutting away vascular structures that was of no interest using a region-of-interest drawing tool operating on axial, sagittal and coronal projections of the vasculature; a procedure that could be performed within minutes for each participant. One operator performed the post processing (CB) and this was performed two times to assess the intra-operator consistency. Furthermore, in a third extraction, we removed all smaller distal arterial branches, secondary to the main distal supply, keeping only the main vascular routes from the most proximal to the most distal end of the vasculature. This control analysis served two purposes. Firstly, it provided means to assess the sensitivity of the derived PWV-values to large variations in manual post-processing. Secondly, it allowed us to investigate the influence that potential kissing arteries, a false junction detected between two arteries that crossed without actually coalescing, had on the PWV-estimation. This was possible since the removal of all but the main paths of the blood supply eliminated such false junctions. Even after this procedure, however, some kisses could still be present in the data in the case that an artery divided into two equally sized large branches that ran in parallel. This was not considered an important issue since in that scenario, the error in the calculated distance should be very small.

A vascular distance algorithm was employed, operating by a step-wise assignment of distances from a user selected seed point in the root of the visible vasculature. The algorithm accomplished two things. Firstly, it provided a distance from the proximal starting points of the internal carotid arteries (ICAs) to all consecutive voxels along the centerlines (using Euclidian distances between centerline points). Secondly, it assured the removal of undesired vessels (since spurs not connecting directly or indirectly to an ICA would not obtain a distance). Since the most proximal centerline voxel (i.e. the voxel closest to the heart) of both left and right ICA functioned as seeds for the distance calculation, each cross-section obtained two distance estimates, due to the connection via the anterior communicating artery of the Circle of Willis. The shorter distance was kept for all cross-sections, as this was believed to be most representative of the distance travelled by the pressure pulse at each location. We note that this could assign a distance to the anterior cerebral arteries which stems from one of the two internal carotid arteries that in actuality was not the main blood flow supplier. Due to the high degree of left-right symmetry of the ICAs and the anterior circulation, we believe that the potential distance error from this simplification is small and it was thus not expected to have an important negative influence on the PWV calculation. Additional anastomoses, beyond that of the circle of Willis, occur at more distal locations in the vasculature,^27^ not detectable with our 4D flow MRI measurements. Therefore, such distal sites of mixing could not interfere with the distance calculation.

### Estimation of pulse wave velocity

**Figure 1.**
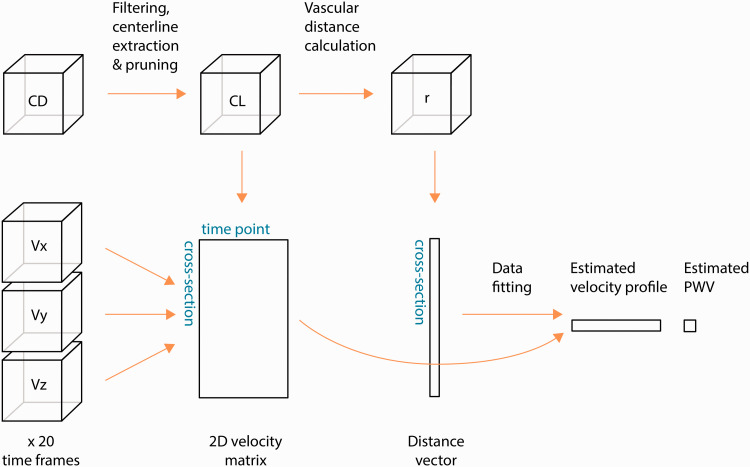
Schematic overview of the process to obtain PWV. The complex difference (CD) image is used to generate a centerline representation of the vascular tree. A user-defined selection determines the centerlines of interest (CL) in which velocity curves, calculated from velocity images, are automatically extracted. The velocity curves are stored in a two-dimensional matrix. A distance calculation is also performed on the CL volume, generating a unique distance (r) for each cross-section that is stored as vector with the same cross-section ordering as the velocity matrix. A model is fitted to the velocity matrix and the distance vector to obtain the PWV as well as the velocity waveform that best explain the observed data.

An overview of the processing steps and the data used in the proposed method is provided in Figure 1. A model of a velocity waveform and phase shifts depending on vascular depth and PWV was fitted to the data. For that, we made a few simplifying assumptions about each measured velocity, $v_{ij},$ measured at the *i*th cross-section in the *j*th time frame and at a distance corresponding to $r_{i}$ from the root of the arterial tree:

1. Each measurement is normally distributed.
2. Disregarding noise, an identical but phase shifted velocity waveform is sampled at each position, at delays given by $r_{i}/\mathrm{PWV}.$ This velocity waveform was denoted as $V\left(t\right).$

In our approach, PWV and V(t) were simultaneously estimated and for that V(t) needed to be parametrized. Here we considered fixed points at time $t_{j}$ , corresponding to the timepoints of the original time frames of the data, forming the vector $V=\left[V\left(t_{1}\right)\;V\left(t_{2}\right)\ldots V(t_{20})\right]$ . Linear interpolation was used to obtain samples of V(t) at arbitrary time points. We write this as $V\left(t\right)=\tilde{V}(t;V)$ where $\tilde{V}(t;V)$ is a linear interpolation function. Using the assumptions above, a maximum likelihood estimator for both the PWV and the velocity profile is given by

$$\mathrm{PWV},\;V=\arg\underset{\mathrm{PWV},V}{\min}\sum_{i=1\;}^{N}W_{i}\sum_{j=1}^{M}{\left(\tilde{V}\left(t_{j}-\frac{r_{i}}{\mathrm{PWV}};V\right){-v}_{ij}\right)}^{2}$$

where M corresponds to the number of timepoints in our 4D flow MRI time series (i.e. M=20 ), *N* corresponds to the total number of cross-sections available to the estimation and $\;t_{j}\;$ corresponds to the time (i.e. in seconds) associated with the *j*th time-frame. $W_{i}$ is a weight factor proportional to the reciprocal of the measurement variance, modeled as function of the scaling applied to each waveform and the ROI area associated with a particular cross-section $\left(W_{i}=\mathrm{area}/{\mathrm{scaling}}^{2}\right)$ . The optimization was performed in Matlab with *fminunc* with a quasi-newton algorithm with numerical gradients. The built-in linear interpolation function *interp1* was used to evaluate $\tilde{V}\left(t;V\right)$ . The initial value for V was set to the average of all measured waveforms while an initial value of 10  m/s was used for the PWV . An initial value of PWV = 40 m/s, or setting the initial value for V to zeros, did not alter the results.

### Statistics

Lilliefors tests were used to investigate normality. Group differences in PWV were assessed using non-parametric, Wilcoxon rank sum tests. Group differences in heart rate was assessed with a student t-test. Pearson correlation was used to assess associations between two variables. All tests were two-sided and p < 0.05 was considered statistically significant.

## Results

**Figure 2.**
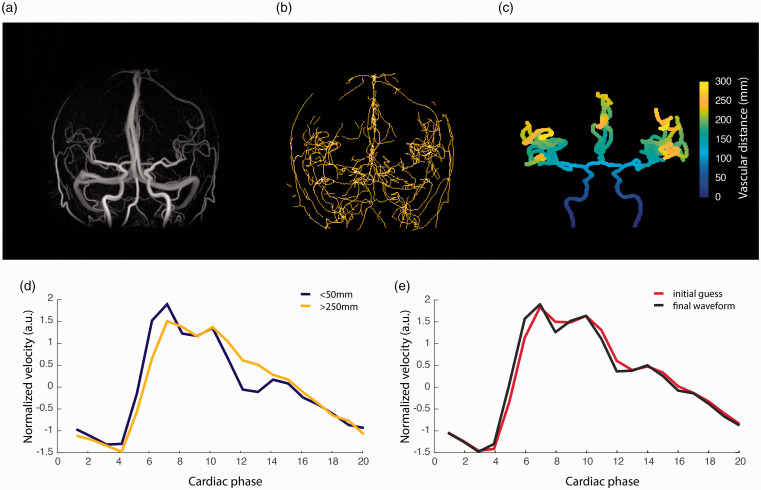
(a) Example angiographic complex difference (CD) image; (b) the raw centerline structure; (c) the corresponding segmented centerline structure (CL) with calculated vascular depth; (d) the average velocity waveforms obtained in the most proximal (depth <50 mm) as well as in the most distal aspects (depth >250 mm) of the visible cerebral vasculature; (e) the initial guess and final estimation of the arterial velocity waveform obtained in the optimization procedure.

Using 4D flow MRI and the proposed processing method (Figure 1), we were able to obtain a global PWV estimate for intracranial arteries for all participants. Figure 2(a) to (c) displays an example angiographic CD image, the centerline structure in its original form as well as the corresponding calculated vascular depth after isolating branches of interest. An illustration of the average velocity waveforms obtained in the most proximal as well as in the most distal aspects of the visible cerebral vasculature, where a small delay can be visually appreciated, are provided in Figure 2(d), exemplifying the feature in the sampled waveforms that allowed estimating PWV. The underlying velocity waveform, as suggested by the optimization procedure to best explain the observed data, is seen in Figure 2(e) together with the initial guess.

**Figure 3.**
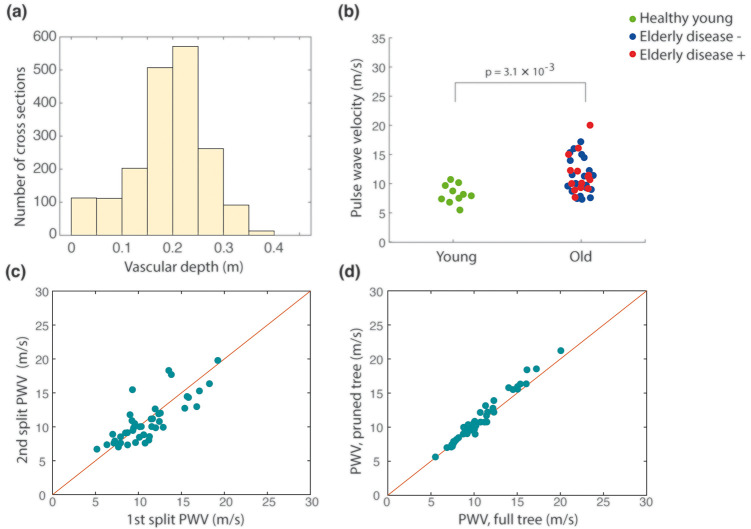
(a) Histogram illustrating the average number of cross-sections available as a function of vascular depth; (b) individual PWV values for young and old participants. Among the elderly, individuals without previous vascular disease, dementia or diabetes (*N* = 21) are marked in blue; (c) stability analysis performed by calculating PWV on half the number of cross-sections; (d) stability analysis performed by calculating PWV using only main routes of vascular supply. The red line in (c) and (d) illustrates perfect agreement.

The number of included cross-sections per distance bin revealed a rapid increase in available cross-sections approximately at a distance corresponding to 15 cm from the root of the vasculature, see Figure 3(a). This corresponds to branching that occurs following the Circle of Willis. At a distance of approximately 25 cm (the distance between the fifth and sixth bin), there was a rapid decrease in number of cross-sections, presumably because at that depth in the vasculature, branches emerged that were too small to be detected by the 4D flow sequence.

Next, we investigated the ability of the method to detect a difference between the elderly and young subjects. The median PWV and interquartile range was 8.1 (7.4–9.7) m/s for the young and 10.7 (9.2–13.6) m/s for the elderly (p = 3.1 × 10^−3^), see Figure 3(b). The maximal vascular distance was 31.4 ± 2.5 cm and 34.6 ± 3.0 cm for the young and elderly, respectively. The heartrate dependent temporal resolution was 47.3 ± 11.2 ms (corresponding to 66.0 ± 12.1 BPM) and 46.1 ± 6.9 ms (corresponding to 66.4 ± 9.5 BPM) for the young and elderly groups respectively (p = 0.91).

In order to specifically address the impact of aging on our intracranial PWV measurements, we analyzed the elderly subsample without previous vascular disease, dementia or diabetes (*N* = 21). This resulted in a median PWV of 10.1 (9.0–14.1) m/s, which was significantly higher than the PWV in the young cohort (p = 1.3 × 10^−2^).

To analyze stability of the estimation we performed a series of control analyses. Firstly, we divided the data from each individual scan into two equally sized halves by sorting every other cross-section (sorted by distance) into two subsets of data for each individual. The internal correlation between the PWVs estimated between the two subsets of data was high (r = 0.80), Figure 3(c). In addition, both subsets’ PWVs were able to detect a difference between the elderly and young groups (p = 3.1 × 10^−3^ and p = 4.3 × 10^−3^, respectively). Second, manual post-processing was performed again to investigate within-operator characteristics. The first and second extraction generated PWV values in excellent agreement (r = 0.99). Finally, a third segmentation where we removed all smaller distal branches, keeping only the main routes of blood flow supply, was generated. On average, this resulted in 33% fewer cross-sections per individual compared to the original extraction. This final extraction produced PWV-values that again were in excellent correspondence with that of the first extraction (r = 0.99) (Figure 3(d)).

## Discussion

We suggest a novel 4D flow MRI method to estimate a single global intracranial PWV designed to be sensitive to general arterial wall stiffening in the cerebral vasculature. The method can be used to advance our understanding regarding the relationship between intracranial vascular changes and brain diseases with a vascular component. The stability of the method, together with the minimal user input required, makes the global intracranial PWV a promising novel biomarker of early cerebrovascular dysfunction.

The problem of finding a PWV in multiple cross-sections was phrased in an optimization framework. This allowed the method to benefit from the substantial amount of data provided by the 4D flow MRI sequence. The number of cross-sections available to the estimation appeared sufficient to derive stable estimates of PWV, as indicated by the data reduction test and removal of smaller distal branches (Figure 3(c) and (d)). Furthermore, the method was capable of detecting a significantly higher PWV for the elderly compared to the young, indicating sensitivity to age-related structural alterations in the walls of intracranial arteries.

Global PWV measurements of distal cerebral vasculature has to our knowledge not been attempted before. However, research has showed an age dependence in PWV measured at the arteries leading up from the neck^28,29^ and ICAs.^18^ A previous study on PWV between the common carotid artery and ICA reported PWVs at 4.7 ± 1.0 m/s for a healthy young (30 ± 3 years, *N* = 23) and 7.9 ± 2.4 m/s for an elderly (62 ± 10 years, *N* = 10) cohort.^29^ Those PWV values were lower than the ones obtained in this study. This could be reasonable considering potential differences in arterial compliance between measurement segments, and that their groups were slightly younger. Another study obtained a median PWV at 17.2 ± 7.4 m/s and 10.0 ± 4.8 m/s for an elderly (73 ± 7 years, *N* = 42) and middle-aged (58 ± 5 years, *N* = 32) group, respectively, measured at a cervical aspect of the ICAs.^18^ Apart from differences in measurement sites and age, some of this discrepancy could stem from biases introduced by the choice of PWV calculation method (e.g., time-shift as the difference between the maximum acceleration points on the systolic upstroke of the flow wave form,^18^ versus the full waveform as utilized within our study). The time shift between measurement sites can be calculated in slightly different ways; time-to-foot, time-to-upstroke, and cross-correlation, to mention a few.^13^ A consensus on the right way to calculate the time shift is yet to be reached. Indeed, different research groups utilize different approaches.^13,18,28,29^ Nevertheless, a study comparing PWV calculated from the systolic upstroke of the velocity waveform compared to utilizing the complete waveform only found small differences.^21^

Transcranial doppler has been used to assess PWV in cranial arteries leading up from the neck. However, intracranial arteries are tortuous making it hard to achieve the right distance measure.^28^ In addition, the technique suffers limitations due to acoustic windows and operator dependence. An alternative approach has been 2D phase contrast MRI, which for example has been used to estimate PWV at the carotid arteries.^29,30^ The downside is that 2D phase contrast MRI is laborious and time consuming, requiring two separate 2D scans to be placed orthogonally to the vessel orientation for each vessel segment. Time resolved 4D flow MRI offers complete brain coverage in all three spatial directions. An advantage is the ease in achieving multiple cross-sections, and that sampling variability in the waveform extraction is reduced due to the synchronized gating between measurement locations.

Measurements of arterial stiffness in central arteries have revealed links to cognitive decline and dementia protein pathology.^14–16,31,32^ Furthermore, research has revealed increased transcranial PWV and pulsatility index for elderly with AD, as well as a clear association between increased transcranial PWV and APOE4 positivity in middleaged.^18^ Moreover, advanced age has been associated with an impaired ability to dampen blood flow pulsatility in the large intracranial vessels.^33^ The proposed PWV measurement could be a useful tool for elucidating the role of intracranial arterial stiffness in these associations. Furthermore, the recently discovered glymphatic system that clears metabolic waste product from the brain^34^ is thought to be powered by pulsatile arterial wall movement,^9^ indicating that PWV assessment can additionally have a role in understanding the glymphatic system.

There are some limitations to the present study. The lack of a reference technique makes it impossible to know true accuracy of the obtained PWVs. In addition, the method required some manual input to crop out vessels of no interest and although the obtained PWVs seemed to be robust to user-related variations in the vascular extraction, the process required a couple of minutes per subject. Arterial “kisses”, falsely indicating a junction between to arteries that cross in proximity of each other, were present in the most straightforward version of the manual segmentation, causing error in the distance of some distal branches that are time-demanding to identify and correct for manually. The effect of such distance errors on the obtained PWV-values was however small as indicated by the control analyses where all smaller distal branches were sacrificed, removing false junctions in the distal vasculature (Figure 3(d)).

A further limitation is the limited sample size and that the age-group comparison made in this paper may not only be representing the effect of age on global intracranial PWV. Even after excluding elderly subjects with neurological and vascular diseases, vascular risk factors were still highly prevalent in the elderly group and could thus have contributed to the large difference in PWV compared to the young participants. A final limitation is the uncertain value in measuring a single brain-wide intracranial PWV. Such a global measure may be diluting local disturbances in isolated arteries or isolated vascular territories. For example, if the distal arteries display the most relevant stiffening from a particular disease perspective, the inclusion of proximal vasculature could obscure meaningful inter-individual differences. In such a scenario, we believe that our method could easily be adapted to measure a PWV in isolated flow territories or in isolated arteries or segments. For example, we envision that the root of the middle cerebral artery (M1) could function as a seed point to analyze pulse wave transmission in the MCA territory. However, such analyses are bound to include shorter vascular distances and we anticipate that, at some point, the time-delay associated with the pulse transmission becomes too small to accurately resolve with the technique.

In conclusion, we present a novel and robust 4D flow MRI method to calculate a global intracranial PWV that utilizes minute temporal shifts in the arterial waveform that arise as the systolic pressure pulse propagates through the branching cerebral vasculature. Using this method, we demonstrate an age-related increase in global PWV of distal intracranial arteries.
